# Predictor bias in genomic and phenomic selection

**DOI:** 10.1007/s00122-023-04479-8

**Published:** 2023-10-25

**Authors:** Hermann Gregor Dallinger, Franziska Löschenberger, Herbert Bistrich, Christian Ametz, Herbert Hetzendorfer, Laura Morales, Sebastian Michel, Hermann Buerstmayr

**Affiliations:** 1 Institute of Biotechnology in Plant Production, Department of Agrobiotechnology, IFA-Tulln, University of Natural Resources and Life Sciences Vienna, Konrad-Lorenz-Str. 20, 3430 Tulln, Austria; 2 Saatzucht Donau GesmbH & Co KG, Saatzuchtstrasse 11, 2301 Probstdorf, Austria

## Abstract

**Key message:**

NIRS of wheat grains as phenomic predictors for grain yield show inflated prediction ability and are biased toward grain protein content.

**Abstract:**

Estimating the breeding value of individuals using genome-wide marker data (genomic prediction) is currently one of the most important drivers of breeding progress in major crops. Recently, phenomic technologies, including remote sensing and aerial hyperspectral imaging of plant canopies, have made it feasible to predict the breeding value of individuals in the absence of genetic marker data. This is commonly referred to as phenomic prediction. Hyperspectral measurements in the form of near-infrared spectroscopy have been used since the 1980 s to predict compositional parameters of harvest products. Moreover, in recent studies NIRS from grains was used to predict grain yield. The same studies showed that phenomic prediction can outperform genomic prediction for grain yield. The genome is static and not environment dependent, thereby limiting genomic prediction ability. Gene expression is tissue specific and differs under environmental influences, leading to a tissue- and environment-specific phenome, potentially explaining the higher predictive ability of phenomic prediction. Here, we compare genomic prediction and phenomic prediction from hyperspectral measurements of wheat grains for the prediction of a variety of traits including grain yield. We show that phenomic predictions outperform genomic prediction for some traits. However, phenomic predictions are biased toward the information present in the predictor. Future studies on this topic should investigate whether population parameters are retained in phenomic prediction as they are in genomic prediction. Furthermore, we find that unbiased phenomic prediction abilities are considerably lower than previously reported and recommend a method to circumvent this issue.

**Supplementary Information:**

The online version contains supplementary material available at 10.1007/s00122-023-04479-8.

## Introduction

The advancement of plant and animal breeding relies on the selection of superior offspring, or superior combinations with enhanced performance, especially increased yield. Historically and even now, this selection has been primarily based on phenotypic observations and pedigree information. The advent of high-throughput genotyping technologies has brought unprecedented insight into the genetic makeup of individuals, thus enabling a selection process based on their genome. Genomic selection (GS) (Meuwissen et al. 2001; Bernardo 1994) is a milestone in applied genetics that builds on more than a century of genetics research (Hickey et al. 2017). It has increased the breeding progress and efficiency in both plant (Crossa et al. 2017; Prasanna et al. 2022) and animal breeding (García-Ruiz et al. 2016) in the last decade. However, due to the cost of genotyping and the limited availability of suitable marker platforms, this technology has not been adopted in some species or breeding programs.

Wheat (*Triticum aestivum* L.) is one of the most important field crops in terms of providing calories and consumable protein to the human diet (Erenstein et al. 2022). Thus, the most important traits in wheat breeding are grain yield (GY) and grain quality (Venske et al. 2019). GY is a highly complex, quantitatively inherited trait and can be dissected into many individually measurable components (e.g., grain size, grains per spike, spikes per area) (Cao et al. 2020), which are strongly modulated by genotype-by-environment (GxE) interactions. These yield components are often, but not always, negatively correlated with each other (Brinton and Uauy 2019; Arriagada et al. 2020; Würschum et al. 2018). Trade-offs do not only exist in terms of the absolute number and dimensions of grains, but also in terms of their chemical and nutritional composition. In wheat, grain starch and grain protein are produced and stored at the same time (Shewry et al. 2012). However, protein deposition requires at least twice the amount of primary energy required for the equivalent amount of starch (Jenner et al. 1991), leading among other factors to a negative phenotypic and genetic correlation between GY and grain protein content (PC) (Kibite and Evans 1984; Simmonds 1995). The protein fraction, especially its content and composition, is a major determinant of end-product quality (Dupont and Altenbach 2003); thus, breeding for grain yield cannot be quality neutral and vice versa. As such, wheat protein content is usually negatively correlated with GY and positively correlated with baking quality. However, the presence of specific alleles of proteins like glutenins or puroindolenes can drastically improve baking quality independently of protein content and have therefore been employed in modern wheat varieties (Würschum et al. 2016).

To mitigate and manage the trade-off between GY and PC described here, a number of strategies have been suggested that allow either selection toward both traits at the same time, or keeping one trait stable while improving the other one. These include the use of protein yield (PY) (Koekemoer et al. 1999; McNeal et al. 1982) or regression residuals in the form of grain yield deviation (GYD) (Rapp et al. 2018; Monaghan et al. 2001). According to Hänsel (2001), regression residuals can be useful if (1) there is a significant inter-cultivar correlation between two quantitative characters; (2) there is a possible causal influence of the “x” on the “y” character; and (3) this possible influence should prove to be physiological. Furthermore, one can develop any kind of selection index by applying weights to both traits or their regression residuals, whereby a particular one is the equal weight protein yield (EWPY) (Rapp et al. 2018). Several studies have shown that these traits and selection indices have a quantitative genetic basis in wheat and other crops (Rapp et al. 2018; Thorwarth et al. 2018; Neuweiler et al. 2021; Geyer et al. 2022) and can be predicted using genetics (Michel et al. 2019).

Field phenotyping has always been a major bottleneck in plant breeding, requiring trained personnel during short time windows in the growing season (Furbank and Tester 2011). The ultimate goal in wheat breeding is to estimate or predict final GY. However, GY is costly and notoriously difficult to measure since it requires replicated trials with large plots to minimize errors due to field heterogeneity or other experimental effects factors. Moreover, the measurement of yield is further complicated by the influence of trial site, local climate and interactions among these factors (Crespo-Herrera et al. 2017). High-throughput phenotyping, ranging from remote sensing using satellites to aerial hyperspectral imaging of single plants has recently been applied in an effort to overcome this bottleneck (Jang et al. 2020; Yang et al. 2017). These data were either combined into empirical vegetation indices (Lai et al. 2018; Palka et al. 2021) from selected spectral frequencies or used in model-based dimensionality reduction techniques (Hansen et al. 2002; Ferrio et al. 2005). An approach which can outperform well-defined vegetation indices in prediction is the use of Bayesian or kernel models on hyperspectral data (Aguate et al. 2017) as well as machine learning approaches (Montesinos-López et al. 2017; Cuevas et al. 2019; Krause et al. 2019; Arya et al. 2022), similar to methods used for GS. Hyperspectral data can furthermore be combined with genomic data using covariates (Rutkoski et al. 2016) or multi-kernel models (Galán et al. 2020).

Since the introduction of near infrared reflectance spectroscopy/spectra (NIRS) in the 1970 s, NIRS has been used to easily and non-destructively derive grain compositional properties as proxies for end-use quality in wheat breeding (Munck 2009) early in the breeding cycle when little seed is available. The high covariance in this spectral information requires special analytical methods like partial least squares regression (PLSR) (Wold et al. 1984). NIRS information can also be used for the characterization of genotype sets based on their phenome or physiochemical fingerprints (Munck 2009). Quality parameters are important in wheat breeding. However, the prime target is to estimate the breeding value for yield, and Ferrio et al. (2004) evaluated the use of milled grain NIRS from several durum wheat (*Triticum turgidum* L. *var. durum*) trials to train PLSR models on GY. They found $r^2$ ranging from 0.04 to 0.33 within and across environments, concluding that prediction of GY from NIRS data is not feasible. However, for a trait with low heritability, low predictive ability is not surprising (Plavšin et al. 2021).

Rincent et al. (2018) combined NIRS of grains with common GS models to predict the yield of a genotype. They called this approach phenomic selection and showed that PS can have predictive ability in the same range as GS. This approach is similar to a method which was recently explored, where genetic markers are replaced or complemented with so-called endophenotypes like transcriptomics (Michel et al. 2021), metabolomics (Schrag et al. 2018) or other omics (Derbyshire et al. 2022) to derive kinship-like relationship matrices. Although the body of literature is growing steadily, only a few studies have investigated phenomics in a similar fashion in major crops. Lane et al. (2020) demonstrated medium to high predictive ability in maize (*Zea mays* L. *ssp. mays*) grain with NIRS-based PS by employing the NIRS-BLUP model. Cuevas et al. (2019) compared kernel methods derived from pedigree, markers or wheat grain NIRS for their usefulness in yield predictions in one environment. The lowest prediction ability was found for NIRS-based kernels, followed by pedigree and marker kernels. Combining markers and NIRS has been found to increase prediction accuracy. Zhu et al. (2021) compared genomic and phenomic predictions in multiple soybean (*Glycine max* L.) populations using the various commonly used GS models. They found high predictive abilities for NIRS, which generally outperformed marker-based predictions for GY and PH. Weiß et al. (2022) compared GS and PS in diverse maize populations for a variety of traits, reporting that PS worked well and outperformed GS when predicting across populations.

As such, the literature suggests that NIRS from grains can be employed beyond their classical use (i.e., the estimation of grain composition) in the form of genomics-like models to predict GY and other traits unrelated to grain composition, such as heading date (HD). Furthermore, PS can potentially outperform GS in terms of predictive ability and is less expensive than genotyping. The mathematical basis for predictive breeding has been laid out by Henderson (1976) in the animal BLUP approach. While the animal best linear unbiased prediction (BLUP) uses pedigree information to estimate the relationship matrix, the genomic BLUP (GBLUP) model (VanRaden 2008) is superior to the animal BLUP approach because it uses the realized relationship matrix which accounts for Mendelian sampling. This realized relationship matrix is trait-independent and unbiased toward specific loci when markers span the genome with sufficient density. PS commonly uses a relationship-like matrix derived from endophenotypes, like hyperspectral reflectance spectra from canopies or grains. In the presence of genetically fixed or environmentally induced correlations of grain compositional traits with the predictor (e.g., in the case of negative GY and PC correlations), the usefulness of NIRS from grain in PS is questionable. Therefore, we used advanced yield trials from an Austrian winter wheat breeding program to investigate GS (using markers) and PS (using NIRS from grains) for GY and other key breeding traits. Furthermore, we investigated genomic and phenomic selection indices with known or empirical underlying correlations, comparing the predictive performance of GS and PS for these traits and potential biases (i.e., conservation of trait correlations) induced by the different predictors (i.e., markers or spectra).

## Materials and methods

### Phenotyping and plant material

We collected phenotypic data from 2019 to 2022 in three locations in Northeastern Austria, routinely as part of the commercial winter wheat breeding program of Saatzucht Donau (Probstdorf, Austria, www.saatzucht-donau.at). Locations WEI and PRO were treated with moderate, split application nitrogen fertilization and without fungicides, according to common agricultural practices in this region. Location DOE was managed according to certified organic practices (https://agriculture.ec.europa.eu/farming/organic-farming_en).

All locations are roughly 150 m above sea level, with mean annual temperatures of $9.7\,^{\circ }\hbox {C}$ and 546 mm rain. Location PRO contains lighter floodplain soils, and WEI and DOE are located close to each other and have Chernozem soil with a relatively higher water storage capacity. Climate at all locations is classified as “Humid Continental Mild Summer, Wet All Year.” Recent years were characterized by persistent spring drought, at times requiring irrigation on some trial locations, depending on soil storage characteristics and local rainfall.

The study was embedded into the routine breeding operation and therefore contained mostly DH or advanced lines in F4:6 or F4:7 stage, in the last two years of testing before official varietal evaluation, as well as check varieties from Saatzucht Donau and other European breeders. The genotypes represent a selection toward high end-use quality for two main markets: early maturing varieties for conventional management in dry continental climate and organic or low input management with good weed suppression as well as exceptional quality characteristics (Muellner et al. 2014; Löschenberger et al. 2008).

We laid out trials as partially replicated (p-rep) designs (Cullis et al. 2006), containing F4:6 and DH4 lines as unreplicated entries and F4:7 and DH lines as well as some registered varieties and crossing parents in two replicates. In total, 939 entries were tested across years. Out of these, 461 lines with complete data in terms of phenotypes (at least GY and PC) as well as NIRS from all three locations and molecular markers were available to evaluate prediction models.

On each plot, we measured grain yield (GY) ($\hbox {dt\,ha}^{-1}$) and estimated grain PC (%) using a custom NIRS calibration. From the genotypic best linear unbiased estimates (BLUEs) on each trial we derived further yield and protein related traits and selection indices. PY ($\hbox {dt\,ha}^{-1}$) was calculated as $PY = GY * PC$. The grain protein deviation (Monaghan et al. 2001) is a well-known method to avoid the negative correlation of PC and GY when selecting for PC. In the GYD the same principle is applied to select for GY. Following the approach of Rapp et al. (2018) and Michel et al. (2019), GYD ($\hbox {dt\,ha}^{-1}$) was derived as the residuals of a linear regression of GY on PC: $GYD = \alpha + \epsilon = gy - \alpha - \beta *{pc}$. EWPY index was calculated, for each environment separately, as the centered ($\mu = 0$) and standardized ($\sigma = 1$) sum of GY and PC: $EWPY = \frac{GY-\mu _{GY}}{\sigma _{GY}} + \frac{PC-\mu _{PC}}{\sigma _{PC}}$ following the method of Rapp et al. (2018) but using the observed GY and PC values rather than the regression residuals.

To assess phenology, we scored plots visually and recorded the day of year when 2/3 of the heads were fully emerged (BBCH 59) as the heading date (HD). HD was recorded in all years at location PRO and recorded in 2020 and 2022 at location DOE, no records for HD are available from WEI. We measured plant height (PH)(cm) as the average distance from topsoil to the tip of the heads on multiple plants per plot between flowering and maturity. PH was recorded in 2020 and 2022 at location WEI, in all years at location PRO and in 2020, 2021 and 2022 at location DOE, leading to a partially complete dataset for HD and PH. In addition we measured the grain quality parameters, thousand kernel/grain weight (TKW)(g) using a seed imaging analyzer (MARViN ProLine, MARViTECH GmbH, Wittenberg, Germany, marvitech.de) and test weight (TW) ($kghL^{-1}$) using volumetric weight determination.

### Phenotypic analysis

To account for spatial variation in the field trials on each location within each year, we used the R-package “sommer” (Covarrubias-Pazaran 2016) to fit linear mixed models for each trait:

1 $$\begin{aligned} y_{ijk} = \mu + g_i + r_j + c_k + sp_{jk} + e_{ijk} \end{aligned}$$

The model in Eq. 1 fits each plot value $y_{ijk}$ with a fixed intercept $\mu$, a genotypic effect $g_i$, a random effect for each row $r_j$ and column $c_k$ as well as a two-dimensional penalized tensor-product of marginal B-Splines (Rodriguez-Alvarez et al. 2017; Welham 2021) $sp_{jk}$, and random residuals $e_{ijk}$. This model was fit, treating genotypes as random to extract genetic and residual variance components ($\sigma ^2_G$, $\sigma ^2_e$) which were in turn used to calculate repeatability estimates according to:

2 $$\begin{aligned} rep^2 = \frac{\sigma ^2_G}{\sigma ^2_G + \sigma ^2_e/{\bar{r}}} \end{aligned}$$

with ${\bar{r}}$ being the effective average replication factor of the p-rep design. The same model in Eq. 1 was fit, treating genotypes as fixed effects, to extract genotypic BLUEs $\hat{g_i}$ for each trial. Genotypic BLUEs were used in the second stage mixed model Eq. 3:

3 $$\begin{aligned} y_{ij} = \mu + g_i + y_j + e_{ij} \end{aligned}$$

as the response $y_{ij}$. This model was fit with a fixed intercept $\mu$, genotypes $g_i$ from all years $y_j$ treated as random effects and random residuals $e_{ij}$ for each location. Genotypes were, again, fit as random to extract genetic and residual variance components ($\sigma ^2_G$, $\sigma ^2_e$) which were this time used to calculate broad-sense heritability estimates (Holland et al. 2003; Schmidt et al. 2019) according to:

4 $$\begin{aligned} H^2 = \frac{\sigma ^2_G}{\sigma ^2_G + \sigma ^2_e/{\bar{r}}} \end{aligned}$$

with ${\bar{r}}$ being the effective average replication factor of genotypes across years (Schmidt et al. 2019). Genotypes were also fit as fixed effects in the same model to extract genotypic BLUEs $\hat{g_i}$ for each location across years.

### Spectra acquisition, aggregation and pretreatment

We acquired NIRS from harvested grains, using a Büchi NIRFlex N-500 (Flawil, Switzerland, www.buchi.com) FT-NIRS with a spectral range of $800\,\hbox {nm}$ to $2500\,\hbox {nm}$, equipped with a NIRFlex Solids measurement cell. Each sample was stored as the average reflectance profile of 32 scans, as wavenumbers from $12500\,\hbox {cm}^{-1}\,\hbox {to}\,4000\,\hbox {cm}^{-1}$ in $4\,\hbox {cm}^{-1}$ increments, yielding 1501 spectral values.

Before further analysis, we normalized each sample ($\mu = 0, \sigma ^2 = 1$) to account for single-light scattering (Barnes et al. 1989; Wadoux et al. 2021). We adjusted plot level spectra from each trial according to the same model as in Eq. 1 to obtain genotypic BLUEs for all wavenumbers within years and subsequently adjusted with the model in Eq. 3 to obtain genotypic spectral BLUEs for each location across years. On the genotypic spectral BLUEs, we applied several filters with the help of the R-package “prospectr” (Stevens and Ramirez-Lopez 2022). We performed “detrend”-filtering with polynomial order $p=2$ and “Savitzky–Golay” filtering with windows size $w=37$, differentiation order $m=1$ (sg1) or $m=2$ (sg2) and polynomial order $p=m+1$.

From column-wise (spectra) and row-wise (genotype) centered ($\mu = 0$) and standardized ($\sigma = 1$) pretreated environment-specific spectra $S_{jk}$ , we built hyperspectral NIRS-derived variance–covariance matrices $H_{jk}$, for each location *j* and each pretreatment *k*. This (hyperspectral) *H* matrix is not to be confused with the concept of a hybrid matrix from markers and pedigrees (Legarra et al. 2009). We derived hyperspectral NIRS-based variance–covariance (relationship-like) matrices from:

5 $$\begin{aligned} H_{jk} = \frac{S_{jk} \times S_{jk}^\prime }{l} \end{aligned}$$

where *l* is the number of predictors (recorded spectral frequencies). This is similar to the method of VanRaden (2008), frequently used for marker-based predictions.

### Genotyping

For genotyping, we sampled F5 or DH2 grains from F4 or DH1 plants. SGS TraitGenetics (Gatersleben, Germany, traitgenetics.com) extracted DNA from single kernels and genotyped using a custom 7k Illumina SNP array yielding 6722 polymorphic markers. The genotyping provider called the alleles, which we recoded on a trinary numeric system with “-1” and “1” being used for either of the homozygous states (aa or AA) and “0” for a heterozygous state (Aa and aA) at a given locus. We imputed missing calls chromosome-wise using the missForest algorithm (Stekhoven and Buehlmann 2012) in R (R Core Team 2023). The final genotypes were filtered for a minor allele frequency of 5%, yielding 5909 filtered markers. The markers capture all 21 chromosomes of wheat, with a density ranging from 58 on chromosome 4D up to 448 on chromosome 3B, therefore saturating the whole genome with sufficient density (Norman et al. 2018). We used the SNP-marker data to generate a marker-based relationship matrix *G*, according to the formula of VanRaden (2008) using the A.mat function implemented in the R-package “sommer” (Covarrubias-Pazaran 2016).

### Prediction models and cross-validations

To evaluate predictive performance, we trained genomic (GBLUP) and phenomic (HBLUP) prediction models according to the same model for various traits:

6 $$\begin{aligned} y = 1_n \mu + g + e \end{aligned}$$

where *y* is the vector of phenotypic BLUEs from across year analysis, $1_n$ is a vector of ones with a fixed intercept $\mu$, random effects *g* of each genotype $g_i$ with distribution $g \sim {\mathcal {N}}(0,\,K \sigma ^{2}_g)$ and the genetic variance $\sigma ^2_g$. Random errors are assumed to follow $e \sim {\mathcal {N}}(0,\,I \sigma ^{2}_e)$ with variance $\sigma ^2_e$. The variance–covariance matrix *K* and subsequently $\sigma ^2_g$ were derived either from markers (*G*) or pretreated NIRS-derived variance–covariance matrices (*H*) from various locations, leading to a set of different models and predictions. Note that *G* only captures genetic differences, whereas *H* is environment-specific and is expected to also capture environmental effect. This might increase the predictive ability of PS if *H* is derived from seeds, especially for traits which describe seed characteristics, like TW or TKW, but possibly also other traits. This can also be undesired if spectra are only available from one environment, but the goal is to predict traits in another environment or predict only the genetic value for a trait.

To assess the predictive ability of the above-mentioned models, we used location-specific across-year BLUEs in a fivefold cross-validation, where the genotypes are randomly split into five equally sized groups, four of which are used for model training and one is used for model validation. The cross-validation procedure was repeated for the same split until every fold was used for validation once. To furthermore assess the effect of training population size, we randomly sampled 100, 200, 300 or 400 lines out of all lines, to perform cross-validation, which was repeated for every size 20 times to get robust parameter estimates. Predictive ability was measured as the Pearson product-moment correlation coefficient of the predicted versus the observed values in the validation fold. Furthermore, we investigated the Pearson correlation of the validation fold’s predicted values with other phenotypes to assess predictor bias.

## Results

### Phenotypic characterization

**Table 1:** Estimated broad-sense heritability of traits on three locations

	GY	PC	GYD	PY	EWPY	HD	PH	TW	TKW
DOE	0.35	0.59	0.31	0.32	0.39	0.88	0.80	0.80	0.79
PRO	0.14	0.48	0.09	0.09	0.23	0.80	0.71	0.68	0.72
WEI	0.34	0.40	0.42	0.49	0.49		0.66	0.66	0.74

Broad-sense heritability $H^2$, estimated from across years analysis per location, for grain yield (GY), protein content (PC), grain yield deviation (GYD), protein yield (PY), equal weight protein yield (EWPY), heading date (HD), plant height (PH), test weight (TW), thousand kernel/grain weight (TKW)

Single-trial repeatability ($rep^2$) was medium to high, ranging from 0.53 to 0.91 for GY and from 0.59 to 0.99 for all other traits in three locations across all four years (Online Resource 1). Broad-sense heritability ($H^2$) (Table 1) was low for GY and medium for PC. Similarly, the indices GYD, PY and EWPY had low to medium heritability. High heritability was observed for HD, PH, TW and TKW.

**Fig. 1:**
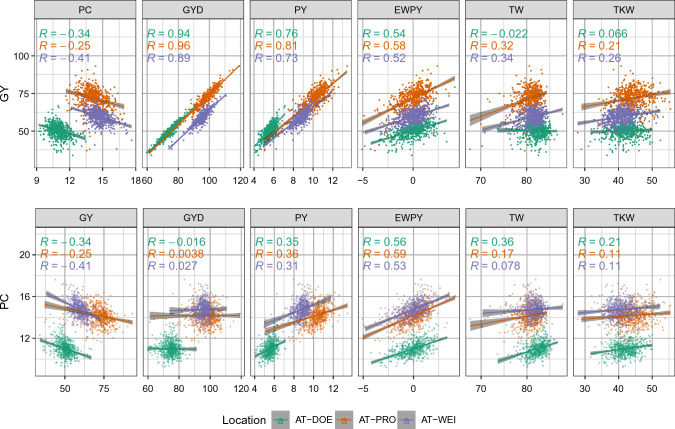
Scatter plots and phenotypic correlations of traits with GY and PC in three environments. Scatter plots for grain yield (GY), protein content (PC), grain yield deviation (GYD), protein yield (PY), equal weight protein yield (EWPY), test weight (TW), thousand kernel/grain weight (TKW) including linear regression line and Pearson’s correlation coefficient *R*

Although broad-sense heritability for GY was low, we observed consistent weak to moderate negative correlations (*R*) between GY and PC in three locations (Fig. 1), with higher negative values in the locations with higher heritability. As expected, given that GY was used to calculate GYD, GYD was highly correlated with GY and did not show any correlation with PC. For PY, we found high positive correlations with GY and moderate positive correlations with PC, indicating that the response in PY is mostly driven by GY. The EWPY index shows balanced correlations to GY and PC, reflecting the parameters which were used to calculate EWPY. In the two conventionally managed environments (PRO and WEI), we observed weak positive correlations of TW and TKW with GY, and in the organic environment, these traits showed weak positive correlations with PC. For HD, we found very weak negative correlations with GY, indicating a slight yield advantage of earliness and no effect on PC. We found the opposite situation for PH, which showed no correlation with GY and weak positive correlations with PC. Phenotypic correlations for all pairwise trait combinations (especially HD and PH with GY and PC) and each location are found in Online Resources 1 and 2.

### NIRS analysis

We fit a mixed model for each spectral wavenumber, using the same models as for each trait. Fitting genotypes as random effects allowed us to extract variance components and calculate repeatability as well as heritability. Fitting genotypes as a fixed effect allowed for the estimation of BLUEs within and across years for each location (Online Resource 1). Spectral repeatability (ranging from $rep^2 =$ 0.39 to 1.0) signatures yielded similar patterns within the same year across different locations, with larger differences between years in the same location, suggesting a stronger year-effect and a weaker location-effect on spectra (Online Resource 2). The phenotypic model did not converge for some wavenumbers, and for these we substituted the BLUEs with the mean of all entries. Likewise, the resulting heritability coefficients for each location across years (ranging from $H^2 =$ 0.338 to 0.762) were very similar across locations (Online Resource 2). Variance components varied widely, with the genotypic variance ranging from $\sigma ^2_g =$ 2.2% to 66.1%, year variance ranging from $\sigma ^2_y =$ 1.4% to 93.7% and residual variance ranging from $\sigma ^2_e =$ 2.4% to 40.9%, suggesting that some spectral regions are highly dependent on the genotype, while others are dependent on year or environment.

### Marker- and NIRS-based relationship matrices

**Fig. 2:**
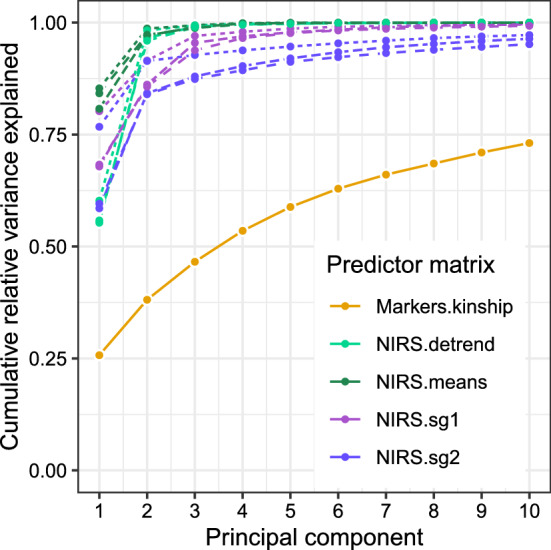
Dimensionality of marker-based matrix (Markers.kinship) and NIRS-based variance–covariance matrices from three locations, visualized using relative cumulative variance of the first 10 principal components. NIRS-based variance–covariance matrices were pretreated using detrending, means (no-pretreatment), first-order (sg1) and second-order (sg2) Savitzky–Golay filtering

We compared the relationship matrices derived from NIRS and the matrix derived from markers against each other using principal component analysis (Fig. 2) and Mantel correlation coefficients (Online Resource 2). Principal component analysis revealed that matrices derived from NIRS required few principal components to explain a substantial part of the variation present ($>90\%$ explained in 5 components or less), but the matrix derived from markers required many more components to explain the same variation ($>90\%$ explained in 35 components). This was also evident from the graphical representations of the kinship matrices (Online Resource 2), which we grouped by hierarchical clustering of the individuals. Here, the NIRS-based relationship matrices show much larger clusters compared to the marker-based matrix, therefore also allowing a relatively finer grouping of genotypes by the GBLUP model and a coarser grouping of genotypes in the HBLUP models. Mantel correlation coefficients between the marker-based kinship matrix and the matrices derived from NIRS were low and ranged from 0.10 to 0.15 depending on location and pretreatment, with the second-order derivative pretreated NIRS matrices showing the highest Mantel correlation coefficients. Mantel correlation coefficients between pretreatments on the same location were high and ranged from 0.78 to 0.93 within the same location. We found medium to low Mantel correlations coefficients across locations, ranging from 0.18 to 0.45 (Online Resource 2).

### Genomic and phenomic prediction ability

**Fig. 3:**
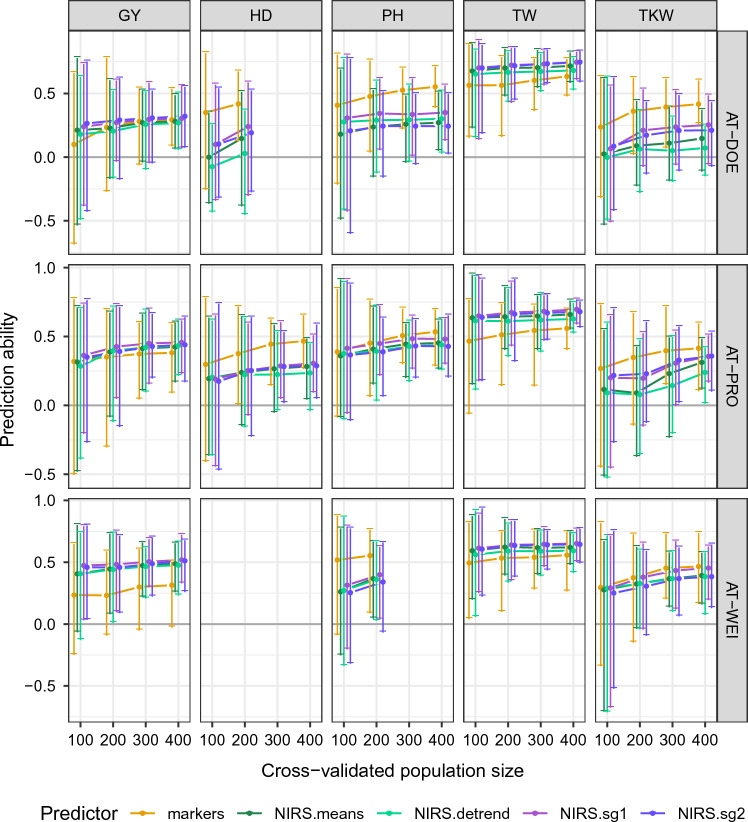
Prediction ability (mean and range) of GBLUP (markers) and HBLUP (NIRS with filter detrend, means, sg1 and sg2) for various traits and cross-validated populations sizes. Prediction ability (r) of GBLUP models, using markers as predictors and HBLUP models, using various pretreated NIRS spectra (detrending, means, first- and second-order Savitzky–Golay filtering) as predictors. Traits under investigation were grain yield (GY), heading date (HD), plant height (PH), test weight (TW) and thousand kernel/grain weight (TKW) predictions evaluated in three environments, DOE, PRO and WEI. Models were evaluated 20 times using fivefold cross-validation, randomly sampling 100, 200, 300 and 400 lines from the whole population for cross-validation. Dots indicate the mean value and whiskers extend to minimum and maximum observed prediction ability

We evaluated marker-based and NIRS-based predictions in three environments for five different traits, including GY, HD, PH, TW and TKW. Prediction ability (*r*) generally increased with population size (Fig. 3), especially for traits with lower overall prediction ability. We observed large variation in prediction ability, with a generally positive trend and lower variability toward larger population size. NIRS-based predictions showed a lower response to increases in population size compared to marker-based predictions. The ranking of marker- or NIRS-based model performance did not clearly change with population size, although for cases in which NIRS outperformed markers (e.g., TW), a re-ranking might have been observed in a larger population due to the stronger response to the population size in the marker-based cross-validations.

We found minor differences in predictive ability of four different NIRS pretreatments. First- and second-order Savitzky–Golay filtering (sg1, sg2) generally performed best, although it was found to be trait dependent. In some location-trait combinations, marker- and NIRS-based predictions yielded very similar average predictive ability while in others, they were clearly separated, showing a better performance of one or the other predictors consistently for a given trait across locations.

In two environments (DOE and PRO), NIRS predictive ability was either similar or slightly higher than marker predictive ability and in the third environment (WEI) NIRS clearly performed better than markers with an average predictive ability ranging from ${\bar{r}} = 0.30 \text{ to } 0.50$ for NIRS and ranging from ${\bar{r}} = 0.25 \text{ to } 0.36$ for markers. (All values for average predictive ability are given in Table S6 in Online Resource 1.)

Due to its high heritability ($H^2 = 0.80 \text{ to } 0.88$), we scored HD in only one location (PRO) for all genotypes and in another location (DOE) for a subset of the genotypes. In both locations, markers served as a substantially better predictor for HD compared to NIRS. Predictive ability of NIRS for HD was low but on average clearly positive, ranging from ${\bar{r}} = 0.22 \text{ to } 0.29$ in PRO. Nevertheless, it is surprising that spectra from grains can predict plant phenology.

Similarly, we scored PH for all genotypes in PRO and for a subset of the genotypes in the other two locations. All predictors showed the potential to predict PH to some extent, but we found a higher predictive ability for markers (ranging from ${\bar{r}} = 0.48 \text{ to } 0.57$) compared to NIRS (ranging from ${\bar{r}} = 0.24 \text{ to } 0.46$) in all locations. In PRO, markers performed only marginally better, while in the other two locations markers clearly outperformed NIRS as a predictor for PH.

Across all traits, we found the highest prediction abilities for TW using markers and NIRS as predictors, with NIRS performing even better than molecular markers in all locations. Prediction ability was already high at the lowest sample size of 100 lines and increased slightly with increasing sample size. The high prediction ability of NIRS as a predictor for TW is not surprising, since it was measured on the same material from which the NIRS data were acquired.

Therefore, it would be reasonable to assume that TKW would also be predictable using NIRS; however, for this trait predictive ability was generally much lower, with markers performing better than NIRS in two locations (DOE and PRO) and similarly in WEI.

To compare predictive performance among the prediction models, we also investigated the use of rrBLUP for markers and NIRS as well as PLSR, GBLUP, and HBLUP for NIRS. The various models yielded similar results, which we therefore only include in Online Resources.

### Predictor bias in genomic and phenomic predictions

**Fig. 4:**
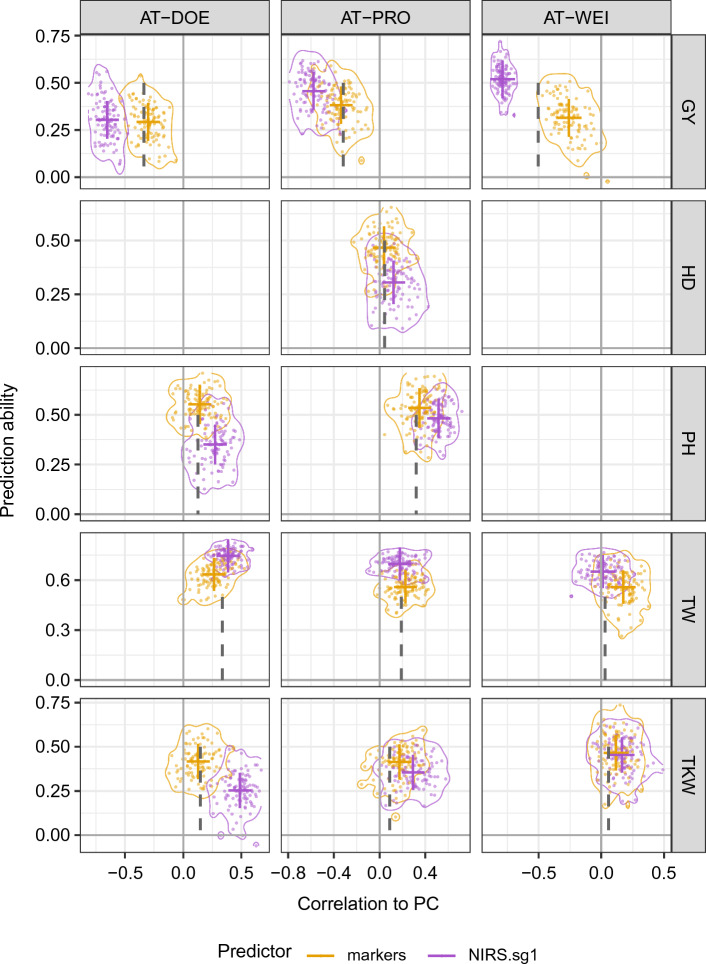
Genomic (GBLUP with markers) and phenomic (HBLUP with NIRS and filter sg1) prediction ability versus bias toward protein content. Genomic (marker-based) and phenomic (NIRS from grains first-order Savitzky–Golay filter) predictions from random sampling of 400 lines in fivefold cross-validations for grain yield (GY), heading date (HD), plant height (PH), test weight (TW) and thousand kernel/grain weight (TKW) in three environments, DOE, PRO and WEI. The vertical axis shows the Pearson correlation of predictions and phenotypic values (prediction ability) in the validation set. The horizontal axis shows the correlation of predictions and PC in the validation set. The dashed line shows the expected value for the correlation to PC, estimated from the phenotypic values of the complete set. Average group values are indicated using solid colored lines in the vertical and horizontal axis. Two-dimensional density lines show probability of 0.95 for the respective group

To address whether markers or NIRS can serve as unbiased predictors, we compared the phenotypic correlations between each trait and PC with the correlations between each trait prediction and PC (Fig. 4). For this analysis, we randomly sampled 400 genotypes from each trait-environment combination. Because of the limited number of genotypes phenotyped for HD and PH, not all environments and population sizes were evaluated in this part of the analysis. For HBLUP predictions, Fig. 4 only shows the first-order Savitzky–Golay filter (sg1), which was the best pretreatment in terms of predictive ability and stability across locations and traits. The results for the other pretreatments are given in Online Resource 2.

Because the negative correlation between GY and PC commonly observed in bread wheat breeding requires independent selection for both traits, this relationship deserves particular attention in phenotypic and genomic selection strategies. In this study, the phenotypic correlations of GY and PC ranged from $r = -0.25$ in PRO to $r = -0.337$ in DOE and up to $r = -0.41$ in WEI. Molecular markers yielded similar (DOE and PRO) or lower correlations between PC and the predictions (WEI), suggesting that markers do not introduce a bias toward protein content. Using various pretreated NIRS to predict GY, we observed predictive ability similar or higher than using markers, although with substantially increased average correlations between trait predictions and PC ranging from $r = 0.51 \text{ to } 0.77$ depending on environment and pretreatment. These results suggest that NIRS can introduce a strong bias toward PC and possibly other grain compositional parameters in the predictions and thereby inflating estimates of prediction ability.

We found no phenotypic correlation between HD and PC in PRO, the only completely evaluated environment for this trait. In this environment, predictions from markers showed no correlation with PC. Predictions from NIRS showed lower predictive ability than from markers, but increased positive correlations with PC. Selecting for earlier HD using NIRS would therefore lead to weak indirect selection toward lower protein content and vice versa.

We scored PH in two environments (DOE and PRO) and found weak positive correlations with PC. In both environments, markers showed higher predictive ability than NIRS, and predictions showed on average the same weak correlations with protein content that we observed in the phenotypic data. However, predictions from NIRS clearly showed increased average correlations with PC independent of pretreatment.

The grain characteristics TW and TKW both showed very weak correlations with PC, with the highest values in DOE, which is the location under organic management conditions. Quite surprisingly, they showed a contrasting response in terms of predictive ability and their correlations with PC. Markers and NIRS were both able to predict TW with high accuracy, although markers performed worse. All predictors reflected the phenotypically observed correlations of TW and PC, except markers in WEI.

The results for TKW were strongly dependent on the trial location. In DOE markers showed high predictive ability and predictions reflected the phenotypic correlations with PC, while NIRS showed lower predictive ability and stronger positive correlations to PC. In the other two locations PRO and WEI, both predictors were overlapping in terms of predictive ability and correlation between predictions and PC, although on average the ranking was similar to DOE.

### Prediction of grain yield and protein content using genomic and phenomic indices

**Fig. 5:**
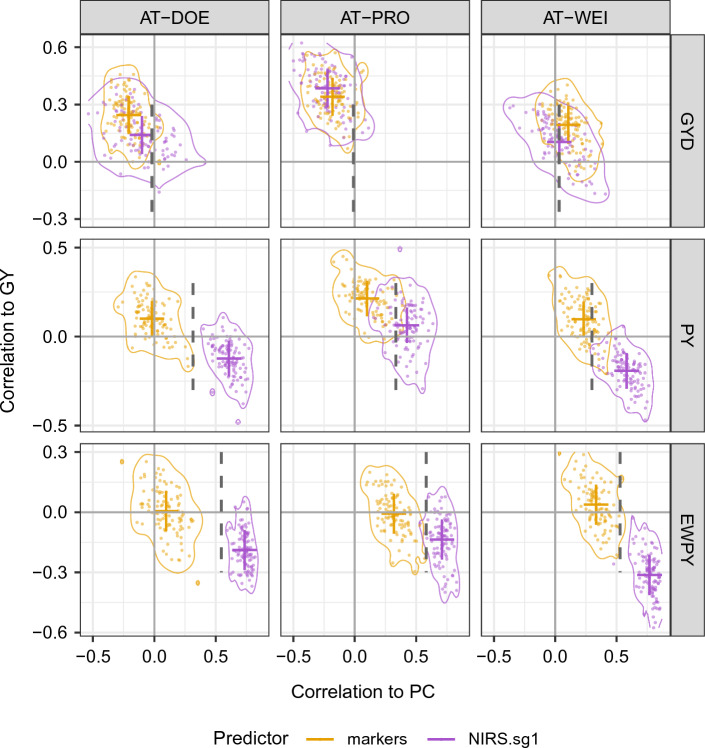
Genomic (GBLUP with markers) and phenomic (HBLUP with NIRS and filter sg1) prediction indices correlation to grain yield (GY) versus correlation to protein content (PC). Genomic (marker-based) and phenomic (NIRS from grains, with first-order Savitzky–Golay filter) index predictions from random sampling of 400 lines in fivefold cross-validations for grain yield (GY), heading date (HD), plant height (PH), test weight (TW) and thousand kernel/grain weight (TKW) in three environments, DOE, PRO and WEI. The vertical axis shows the Pearson correlation of predictions and grain yield (GY) phenotypic values in the validation set. The horizontal axis shows the Pearson correlation of predictions and protein content (PC) values in the validation set. Dashed lines show the expected value for the correlation to GY and PC, estimated from the phenotypic values of the complete set. Average group values are indicated using solid colored lines in the vertical and horizontal axis. Two-dimensional density lines show probability of 0.95 for the respective group

For selection indices, prediction ability is not the main interest, but rather correlations between the index of interest and the traits the index was derived from (Fig. 5). Selection indices are designed to show either no or a well-defined correlation with their basis traits. Therefore, we used selection indices (GYD, PY, EWPY) to address whether phenomic prediction for GY was biased toward PC and if said bias could be mitigated using genomic or phenomic selection indices.

GYD is designed to be uncorrelated with PC, while being highly positively correlated with GY. Prediction ability for GYD using markers was low, ranging from 0.15 to 0.29 across locations, but in most cases even lower using NIRS as predictors, ranging from 0.02 to 0.30 across locations and pretreatments (Online Resource 1). GYD was weakly positively correlated with GY for both markers and NIRS on average; only in location PRO was the average correlation between GYD and GY moderate, exceeding 0.3 for all predictors. Although we expected to observe no correlation between predicted GYD and PC, we found low negative or positive correlations ranging from $-$0.229 to 0.112 across predictors and locations, most likely caused by residual correlations in the phenotypic estimates of GYD, which had not been accounted for.

PY is a type of environmentally dependent multiplicative index with variable correlations with its basis traits. In the three locations, PY showed high positive correlations with GY and weaker positive correlations with PC, therefore putting a much larger weight on GY than PC. The prediction abilities ranged from 0.08 to 0.27 across locations using markers and from 0.14 to 0.35 using NIRS as predictors across locations and pretreatments. The correlations between PY and its basis traits revealed that marker-based predictions were positively correlated to GY in all environments, ranging on average from 0.08 to 0.2. Furthermore, marker-based predictions of PY either had no or weak positive correlation with PC. Predictions derived from various pretreated NIRS showed very low positive or low negative correlations with GY on average, ranging from $-$0.19 to 0.077. Contrary to our expectations, correlations between PY predictions from NIRS and PC were similar to their respective phenotypic correlations, ranging from 0.17 to 0.36 in PRO, or showed substantially higher correlations, ranging from 0.17 to 0.59 in the other locations.

EWPY is designed to show balanced correlations with its basis traits. Using markers as predictors, we found similar prediction ability as for the other indices, ranging from 0.09 to 0.37 across locations. Using NIRS as predictors, we found the highest prediction ability across all indices, ranging from 0.29 to 0.50 across locations and pretreatments. The correlations between EWPY and its basis traits were similar to that of the PY index, with low or negative average correlations of predictors with GY for markers and NIRS, respectively, and a bias (over-correlation) toward PC using NIRS as predictors.

## Discussion

Estimating breeding values facilitates efficient selection of breeding candidates. Multi-location, multi-year replicated trials can allow very accurate estimation of phenotypic values, sometimes referred to as “true” breeding values, but such trials are expensive and only employed in later stages of a breeding program. However, the breeding values of individuals can be estimated from related individuals with known breedings value much earlier in the breeding cycle, although with some uncertainty. In the well-established concept of GS, molecular markers are used as predictors, establishing covariance (relatedness) between individuals. In this study, we investigated the use of endophenotypes instead of molecular markers for prediction of breeding values in a wheat breeding program, for three environments in Austria, which was also termed genomics-like omics-based (GLOB) selection (Robert et al. 2022). This type of PS is performed using NIRS from wheat grains as a predictor.

The negative correlation between GY and PC is a well-known phenomenon in wheat breeding and was also observed in this study. Selection on only one of these traits can lead to indirect selection on the other trait, and is therefore inefficient. A variety of methods have been derived to overcome this, some of which we also investigated in this study, to show whether they can also be used in combination with PS.

### Relationship matrices

Several pretreatments to the NIRS data have been evaluated to find the best performing pretreatment. Although first- and second-order derivatives performed well in many cases, we were not able to clearly identify an optimal pretreatment. Finding the best pretreatment for the use of NIRS in PS has not been investigated in the existing literature (Robert et al. 2022; Rincent et al. 2018; Lane et al. 2020; Zhu et al. 2021). NIRS pretreatment optima may need to be identified on a trait-, crop-, and breeding program-specific basis, as is common for other areas of chemometrics (Buddenbaum and Steffens 2012). We aimed to answer this question by comparing variance–covariance (relationship) matrices derived from markers and NIRS using principal component analysis and Mantel correlation analysis. First- and second-order derivatives showed the highest Mantel correlation coefficients to the kinship matrix derived from markers, although the differences across pretreatments were minor. Principal component analysis of the various kinship matrices revealed that the matrices derived from NIRS required substantially fewer principal components to explain the majority of the variation present. This is also visible in the graphical representations of the kinship matrices, showing lower dimensionality for the NIRS-based matrices compared to the marker-based matrix.

### Phenomic prediction ability

The size and composition of the training set is of major importance in optimizing the accuracy of GS (Fernández-González et al. 2023). To address whether this was also the case for PS, we performed random sampling of different cross-validation set sizes and estimated the increase in prediction ability with increasing set size. Our results were trait dependent to some extent. However, in cases where PS showed similar or higher prediction ability compared to GS, the increase in prediction ability with larger training set size was lower with PS compared to GS, suggesting that smaller training sets are sufficient for PS. This suggest that PS could be of particular interest to smaller breeding programs, where the larger training sets required for GS may be lacking.

We investigated the feasibility of PS for several traits of very different genetic architecture and agronomic importance. GY is the main target in wheat breeding and it has been established that GY is a trait with very complex genetic architecture and minor effects distributed across the genome. For GY, we found that PS performed similar or better than GS in three environments. HD, PH, TW and TKW have a simpler genetic architecture than GY. HD and PH are important for identifying genotypes suitable for specific growing conditions, regions or management practices. For these traits, we found that GS performed better than PS. Surprisingly, PS showed some predictive ability for these traits, although there is no clear physiological connection between grain composition and these traits. It is, however, likely that the correlations between NIRS and HD/PH have been the result of selection for specific markets (organic/conventional) within the material we used for this study (Löschenberger 2022, pers. comm.). For example, the weak positive correlation between PH and PC in this study may reflect breeders’ selection for lines suitable for organic production systems. These require excellent baking quality, and therefore high PC, as well as strong competitiveness against weeds. For the quality traits TW and TKW, we found contrasting results, although they were both measured on the same material used for NIRS. Therefore, we assume that NIRS would be a good predictor for these traits. For TW, both markers and NIRS performed well, but PS consistently outperformed GS. TKW was generally harder to predict than TW and with GS outperforming PS in most cases. Rincent et al. (2018) found that PS clearly outperformed GS for GY and HD if spectra from the same environment were used, but the superiority of PS over GS was not as clear if spectra from different environments were used. Using soybean canopy spectra, Zhu et al. (2021) found that PS outperformed GS for traits with complex genetic architecture, while GS was more predictive than PS and for traits with simpler genetic architecture (i.e., governed by few genetic loci). In a similar study using spectra from grains, Zhu et al. (2021) found that PS outperformed GS for GY and PH, but performed worse for TKW. The variability in rankings between models and traits found here versus previous studies complements the results from Weiß et al. (2022), who found that ranking depends to some extent on the population under investigation.

### Correlations of predictions

According to the central dogma of molecular biology, the expression of genes leads to an observable phenotype, which is modulated by the environment as well as genotype-by-environment interactions. Molecular markers can capture genetic relationships between plants, which can be employed by GBLUP to predict plant phenotypes of unobserved genotypes. If the molecular markers are distributed at sufficient density and uniformity across the genome, capturing most of the genetic diversity that influences trait variation in the population, the relationship between different traits (i.e., correlations) should also be preserved in the predictions. Therefore, we expect molecular markers to be unbiased predictors. NIRS are highly tissue specific; however, according to the literature, relationships derived from NIRS seem to be useful in predicting plant phenotypes. In wheat breeding, NIRS are commonly used to estimate the protein content of seeds with high accuracy. However, it is necessary to select for multiple traits independently at the same time, especially for GY and PC.

The common way to judge the performance of a genomic or phenomic prediction model is prediction ability or accuracy, as derived from k-fold cross-validation within or validation across environments, years or populations. The relationship between predictions and other traits is, however, mostly ignored. In wheat breeding, the relationship between GY and PC is of particular importance. Therefore, we investigated the correlations of GY and other traits predictions to PC and compared these to the phenotypically observed correlations. For GY we observed that GS predictions were largely replicating the phenotypically observed correlations to PC while PS showed severe negative “over-correlation” or bias toward PC. This means that the use of PS for GY would lead to unintentional selection toward low PC. This is for most breeding programs not only undesirable, it is also not sustainable since a biological limit in seed PC could be reached within few generations (Moose et al. 2004), rendering PS ineffective. One of the first studies trying to predict GY from NIRS of milled grains (Ferrio et al. 2004) reported the correlations of measured and predicted GY from PLSR to several other seed components. The measured GY showed medium to low correlation with seed nitrogen content (which is highly predictive for protein content), predicted grain yield showed a high negative correlation with seed nitrogen content, similar to our results. For HD and PH we found a similar bias with lower absolute magnitude, while the correlations of TW and TKW predictions with PC did not show a clear trend.

### Prediction of phenomic and genomic indices

An important question that remains from the findings on predictor bias is whether there is any predictive ability in PS for GY, beyond the exploitation of the negative correlation between GY and PC. To answer this question, we investigated the use of three different selection indices in combination with GS and PS and evaluated their usefulness, which has not been done before to our knowledge. Although both GS and PS were somewhat predictive of these indices a key issue is whether the prediction indices are correlated with the traits that make up the index, namely GY and PC. The genomic selection index for GYD showed positive correlations with GY in all locations. The phenomic selection index for GYD predictive performance was heterogeneous across locations, performing equally well in one location and severely worse in the two other locations. Genomic and phenomic indices showed low negative correlations with PC in two locations, and this underestimation may have been due to environmental masking of the genetically determined negative correlations. Genomic PY and EWPY indices showed no or very low positive correlation with GY and PC, suggesting that the simultaneous selection for both traits may not be feasible, but this may have also been impeded by inaccurate estimation of the underlying genetic relationship. For the phenomic PY and EWPY index evaluation, bias to PC and negative correlations with GY were observed, suggesting that indices containing both traits require much stronger weights for GY compared to PC to be efficient. As reported by other studies (Michel et al. 2019; Rapp et al. 2018), genomic selection indices have additional merit compared to independent selection on both traits and the choice of weights seems to be even more critical for phenomic indices.

The choice of using the GBLUP model for prediction of phenotypes from NIRS did not seem to provide additional prediction power compared to PLSR, which is well established in chemometrics. Furthermore, GY predictions from NIRS using PLSR suffered from the same bias toward PC as predictions from the GBLUP model, suggesting that this is not a property of the prediction model but one of the predictor.

Molecular markers are measured once and are constant across environments. Although NIRS spectra are already used for the accurate estimation of seed composition in most breeding programs and are therefore essentially available without additional cost, their usefulness in prediction of seemingly unrelated traits like yield should be evaluated. Furthermore, to reduce sampling biases in endophenotypes, decisions must be made about which environments or even plots to acquire these from, or how to combine and aggregate them if taken from multiple plots, trials or years. While endophenotypes such as NIRS are environment or even plot-specific and can pose new challenges, they might also provide the opportunity to disentangle environmental and genetic influences, rather than simply averaging across environmental effects.

Our results are largely relevant to winter bread wheat breeding, where grain protein content is a key trait. In other crops such as maize and soybean, grain components like water content (influenced by maturity class) or oil content (negatively correlated with yield) might be additional factors which enable indirect prediction ability of NIRS spectra from seeds. For example in soybean, the negative correlation between yield and seed protein content has also been reported (Orf et al. 1999), but this is confounded by the fact that seed protein and oil content are also inversely correlated due to competition for nutrient allocation or pleiotropic quantitative trait loci (QTLs) (Patil et al. 2017; Chung et al. 2003). Duhnen et al. (2017) reported a medium negative correlation between seed yield and protein content in late maturing lines and no correlation in earlier lines. These correlations do not necessarily have a simple genetic basis, but could also be induced by interactions with the environment.

## Conclusions

Recently published literature suggests that PS is a promising alternative or complement to GS in wheat, soybean or maize breeding as well as in other crops. In many cases, PS seems to outperform GS. Our results suggest that prediction abilities for PS might be over-estimated and that this over-estimation in PS prediction ability may be based on correlations between GY and grain compositional parameters, which could then lead to unintentional or undesirable indirect selection on these parameters. Because previous studies did not provide estimates for grain composition, it is challenging to address whether the prediction abilities reported were over-estimated and whether predictions led to biases or exhibited distorted correlations. We also used selection indices to show that this bias can be quite severe, and the weights the index was designed for might not be retained. Indices with a high weight for GY like GYD might still be useful for overcoming the predictor bias in PS, although we were not able to clearly show this, probably due to limited cross validation set size. Hence, for the moment, we recommend regarding the usage of PS for traits like grain yield with great care and to take correlations with grain compositional traits like PC into account by using selection indices in order to avoid an undesirable indirect selection for these parameters.

## Supplementary Information

Below is the link to the electronic supplementary material.

**Supplementary Materials:** S1: Trait statistics of locations within years. S2: Trait statistics of locations across years. S3: Phenotypic correlations of all traits on all locations. S4: NIRS statistics of locations within years. S5: NIRS statistics of locations across years. S6: Marginal means estimated from a linear model with prediction ability, correlation prediction to protein content or grain yield as the response, and model, trait, location and predictor as fixed effects as well as log(number of samples) as a covariate, including all possible multi-way interactions.(pdf 1,585KB)

**Supplementary Materials:** Phenotypic trait correlations. Normalized adjusted spectra, repeatability and variance components within years. Normalized adjusted spectra, heritability and variance components across years. Mantel correlation of kinship and NIR derived similarity matrices and variance explained by principal components from these matrices. Visualized kinship matrix from markers. Visualized kinship matrices derived from various (locations and pretreated) NIR. Prediction ability of GBLUP, HBLUP and PLSR for all traits, indices and locations. Prediction ability vs prediction correlation to PC of GBLUP, HBLUP and PLSR for all traits and locations. Prediction ability vs prediction correlation to PC of GBLUP, HBLUP and PLSR for all indices and locations. (pdf 35,209KB)

## Data Availability

The datasets generated and/or analyzed during the current study are available from the corresponding author on request. Analyses were conducted using R version 4.2.2 (R Core Team 2023). R packages included “missForest” version 1.5 (Stekhoven and Buehlmann 2012), “prospectr” version 0.2.7 (Rodriguez-Alvarez et al. 2017) and “sommer” version 4.2.0 (Covarrubias-Pazaran 2016).

## Abbreviations

BLUE: Best linear unbiased estimate
BLUP: Best linear unbiased prediction
DOE: Dörfles
EWPY: Equal weight protein yield
GBLUP: Genomic BLUP
GLOB: Genomics-like omics based
GS: Genomic selection
GY: Grain yield
GYD: Grain yield deviation
HBLUP: Hyperspectral BLUP
HD: Heading date
NIRS: Near-infrared reflectance spectroscopy/spectra
PC: Protein content PH plant height
PLSR: Partial least squares regression
PRO: Probstdorf
PS: Phenomic selection
PY: Protein yield
QTL: Quantitative trait locus
rrBLUP: Ridgeregression BLUP
TKW: Thousand kernel/grain weight
TW: Test weight
WEI: Weikendorf
