# Correction: Predictor bias in genomic and phenomic selection

**DOI:** 10.1007/s00122-024-04611-2

**Published:** 2024-04-25

**Authors:** Hermann Gregor Dallinger, Franziska Löschenberger, Herbert Bistrich, Christian Ametz, Herbert Hetzendorfer, Laura Morales, Sebastian Michel, Hermann Buerstmayr

**Affiliations:** 1 Institute of Biotechnology in Plant Production, Department of Agrobiotechnology, IFA-Tulln, University of Natural Resources and Life Sciences Vienna, Konrad-Lorenz-Str. 20, 3430 Tulln, Austria; 2 Saatzucht Donau GesmbH & Co KG, Saatzuchtstrasse 11, 2301 Probstdorf, Austria

**Correction to: Theoretical and Applied Genetics (2023) 136:235** 10.1007/s00122-023-04479-8

It has come to our attention that the original version of this article contained errors in the formatting of number ranges. Specifically, number ranges were inadvertently presented without the standard range indicator (e.g., "–" or "to"), and were instead written without any space between numbers, leading to potential confusion.

Examples of the incorrect formatting are as follows:

- Introduction Section: "0.040.33" should be "0.04 to 0.33".
- Results Section: "0.530.91" should be "0.53 to 0.91".
- Throughout the text, similar instances of number range formatting errors occur.

We sincerely apologize for these mistakes and any confusion they may have caused. The errors have been corrected in the online version of the article. We thank our readers for their understanding and the scientific community for their support.

For any further information or clarification, please contact the corresponding author at hermann.dallinger@boku.ac.at

