# Supplementary material for: Predictor bias in genomic and phenomic selection
Source: Theor Appl Genet. 2023 Oct 25;136(11):235. doi: 10.1007/s00122-023-04479-8 (PMC10600307; doi:10.1007/s00122-023-04479-8)
Supplement: Supplementary file 1 — Supplementary Materials: S1: Trait statistics of locations within years. S2: Trait statistics of locations across years. S3: Phenotypic correlations of all traits on all locations. S4: NIRS statistics of locations within years. S5: NIRS statistics of locations across years. S6: Marginal means estimated from a linear model with prediction ability, correlation prediction to protein content or grain yield as the response, and model, trait, location and predictor as fixed effects as well as log(number of samples) as a covariate, including all possible multi-way interactions.(pdf 1,585KB) [file 122_2023_4479_MOESM1_ESM.pdf]

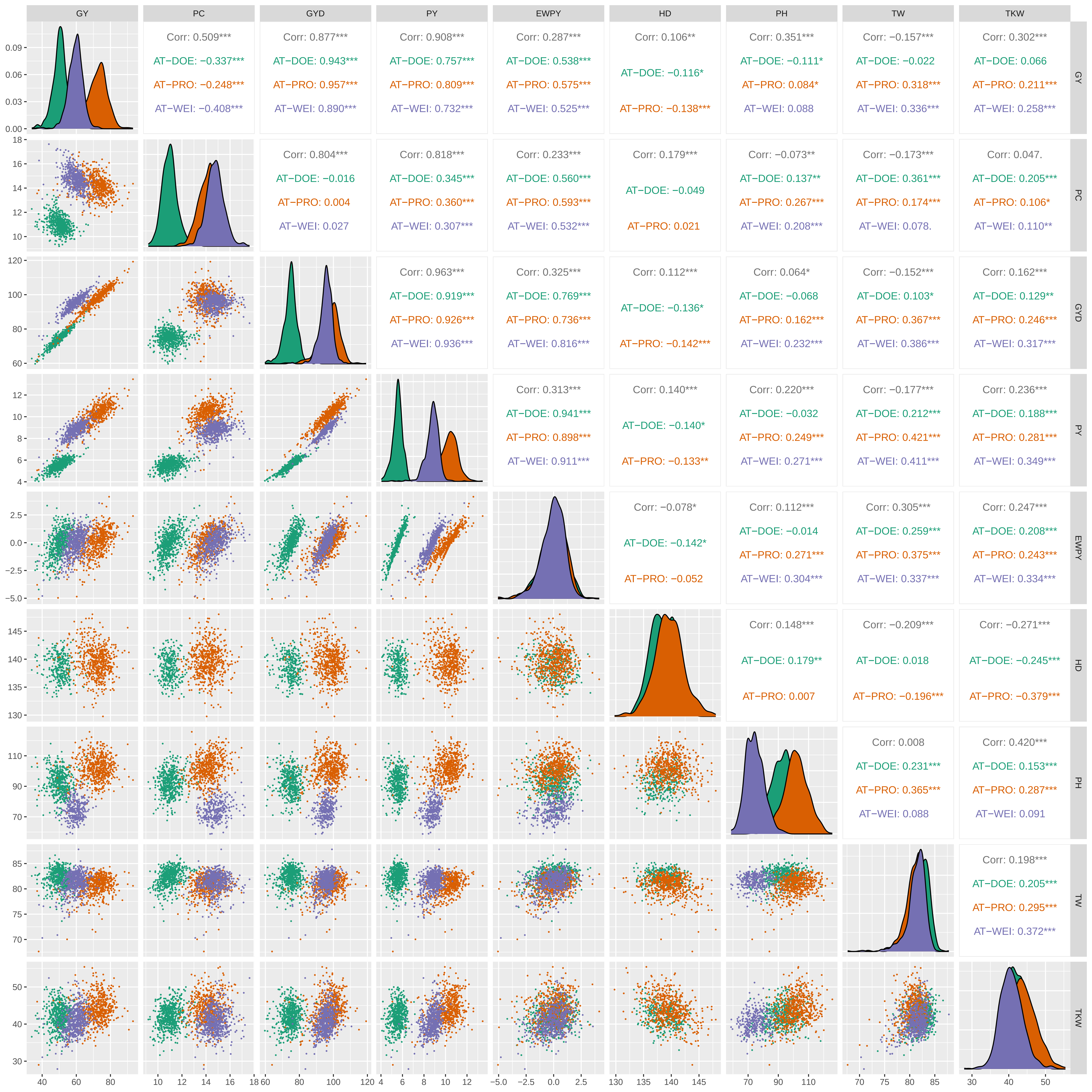
GY
PC
GYD
PY
EWPY
HD
PH
TW
TKW
Corr: 0.509***
AT−DOE: −0.337***
AT−PRO: −0.248***
AT−WEI: −0.408***
Corr: 0.877***
AT−DOE: 0.943***
AT−PRO: 0.957***
AT−WEI: 0.890***
Corr: 0.908***
AT−DOE: 0.757***
AT−PRO: 0.809***
AT−WEI: 0.732***
Corr: 0.287***
AT−DOE: 0.538***
AT−PRO: 0.575***
AT−WEI: 0.525***
Corr: 0.106**
AT−DOE: −0.116*
AT−PRO: −0.138***
Corr: 0.351***
AT−DOE: −0.111*
AT−PRO: 0.084*
AT−WEI: 0.088
Corr: −0.157***
AT−DOE: −0.022
AT−PRO: 0.318***
AT−WEI: 0.336***
Corr: 0.302***
AT−DOE: 0.066
AT−PRO: 0.211***
AT−WEI: 0.258***
Corr: 0.804***
AT−DOE: −0.016
AT−PRO: 0.004
AT−WEI: 0.027
Corr: 0.818***
AT−DOE: 0.345***
AT−PRO: 0.360***
AT−WEI: 0.307***
Corr: 0.233***
AT−DOE: 0.560***
AT−PRO: 0.593***
AT−WEI: 0.532***
Corr: 0.179***
AT−DOE: −0.049
AT−PRO: 0.021
Corr: −0.073**
AT−DOE: 0.137**
AT−PRO: 0.267***
AT−WEI: 0.208***
Corr: −0.173***
AT−DOE: 0.361***
AT−PRO: 0.174***
AT−WEI: 0.078.
Corr: 0.047.
AT−DOE: 0.205***
AT−PRO: 0.106*
AT−WEI: 0.110**
Corr: 0.963***
AT−DOE: 0.919***
AT−PRO: 0.926***
AT−WEI: 0.936***
Corr: 0.325***
AT−DOE: 0.769***
AT−PRO: 0.736***
AT−WEI: 0.816***
Corr: 0.112***
AT−DOE: −0.136*
AT−PRO: −0.142***
Corr: 0.064*
AT−DOE: −0.068
AT−PRO: 0.162***
AT−WEI: 0.232***
Corr: −0.152***
AT−DOE: 0.103*
AT−PRO: 0.367***
AT−WEI: 0.386***
Corr: 0.162***
AT−DOE: 0.129**
AT−PRO: 0.246***
AT−WEI: 0.317***
Corr: 0.313***
AT−DOE: 0.941***
AT−PRO: 0.898***
AT−WEI: 0.911***
Corr: 0.140***
AT−DOE: −0.140*
AT−PRO: −0.133**
Corr: 0.220***
AT−DOE: −0.032
AT−PRO: 0.249***
AT−WEI: 0.271***
Corr: −0.177***
AT−DOE: 0.212***
AT−PRO: 0.421***
AT−WEI: 0.411***
Corr: 0.236***
AT−DOE: 0.188***
AT−PRO: 0.281***
AT−WEI: 0.349***
Corr: −0.078*
AT−DOE: −0.142*
AT−PRO: −0.052
Corr: 0.112***
AT−DOE: −0.014
AT−PRO: 0.271***
AT−WEI: 0.304***
Corr: 0.305***
AT−DOE: 0.259***
AT−PRO: 0.375***
AT−WEI: 0.337***
Corr: 0.247***
AT−DOE: 0.208***
AT−PRO: 0.243***
AT−WEI: 0.334***
Corr: 0.148***
AT−DOE: 0.179**
AT−PRO: 0.007
Corr: −0.209***
AT−DOE: 0.018
AT−PRO: −0.196***
Corr: −0.271***
AT−DOE: −0.245***
AT−PRO: −0.379***
Corr: 0.008
AT−DOE: 0.231***
AT−PRO: 0.365***
AT−WEI: 0.088
Corr: 0.420***
AT−DOE: 0.153***
AT−PRO: 0.287***
AT−WEI: 0.091
Corr: 0.198***
AT−DOE: 0.205***
AT−PRO: 0.295***
AT−WEI: 0.372***

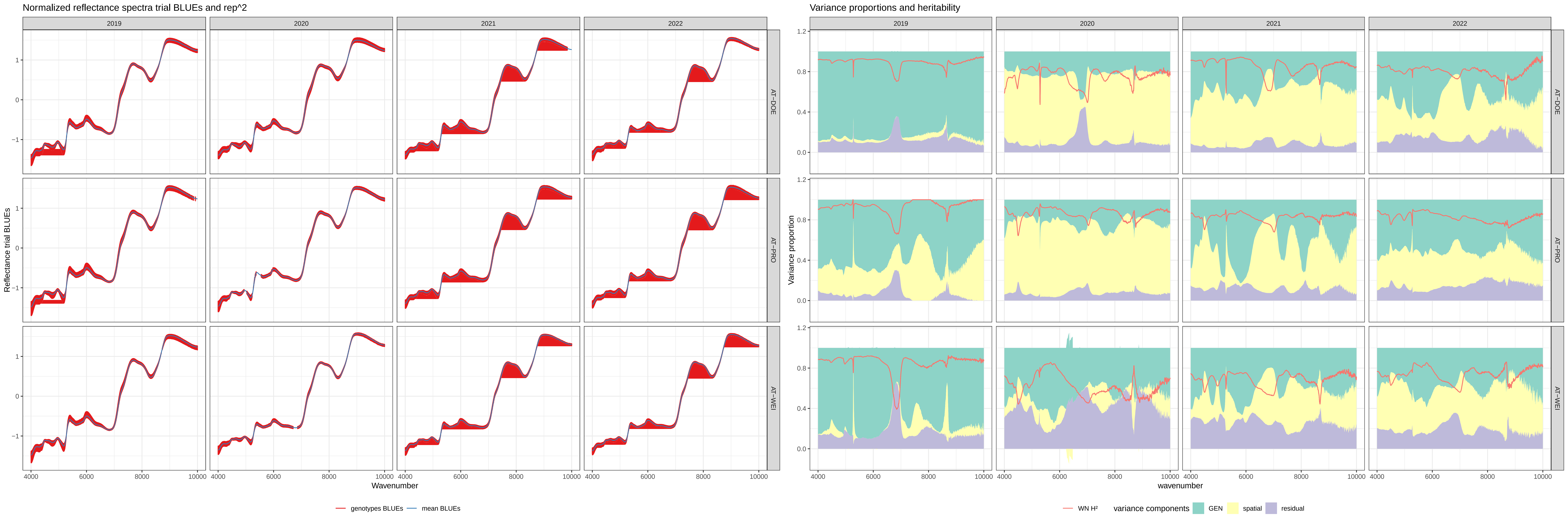

Normalized reflectance spectra trial BLUEs and rep^2
2019
2020
2021
2022
AT−DOE
AT−PRO
AT−WEI
Reflectance trial BLUEs
Wavenumber
genotypes BLUEs
mean BLUEs
Variance proportions and heritability
Variance proportion
wavenumber
WN H²
variance components
GEN
spatial
residual

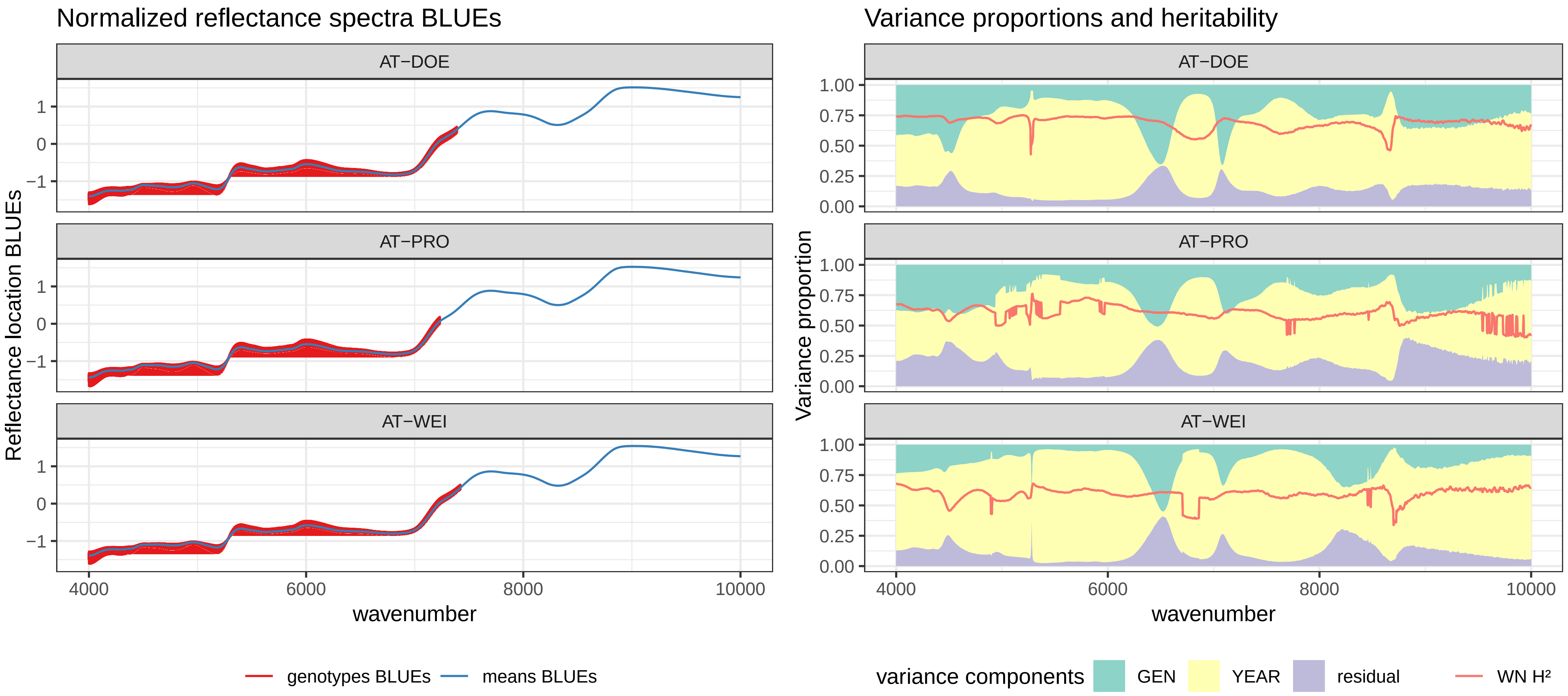

Normalized reflectance spectra BLUEs
Variance proportions and heritability
AT−DOE
AT−PRO
AT−WEI
Reflectance location BLUEs
Variance proportion
1
0
−1
1.00
0.75
0.50
0.25
0.00
4000
6000
8000
10000
wavenumber
genotypes BLUEs
means BLUEs
variance components
GEN
YEAR
residual
WN H²

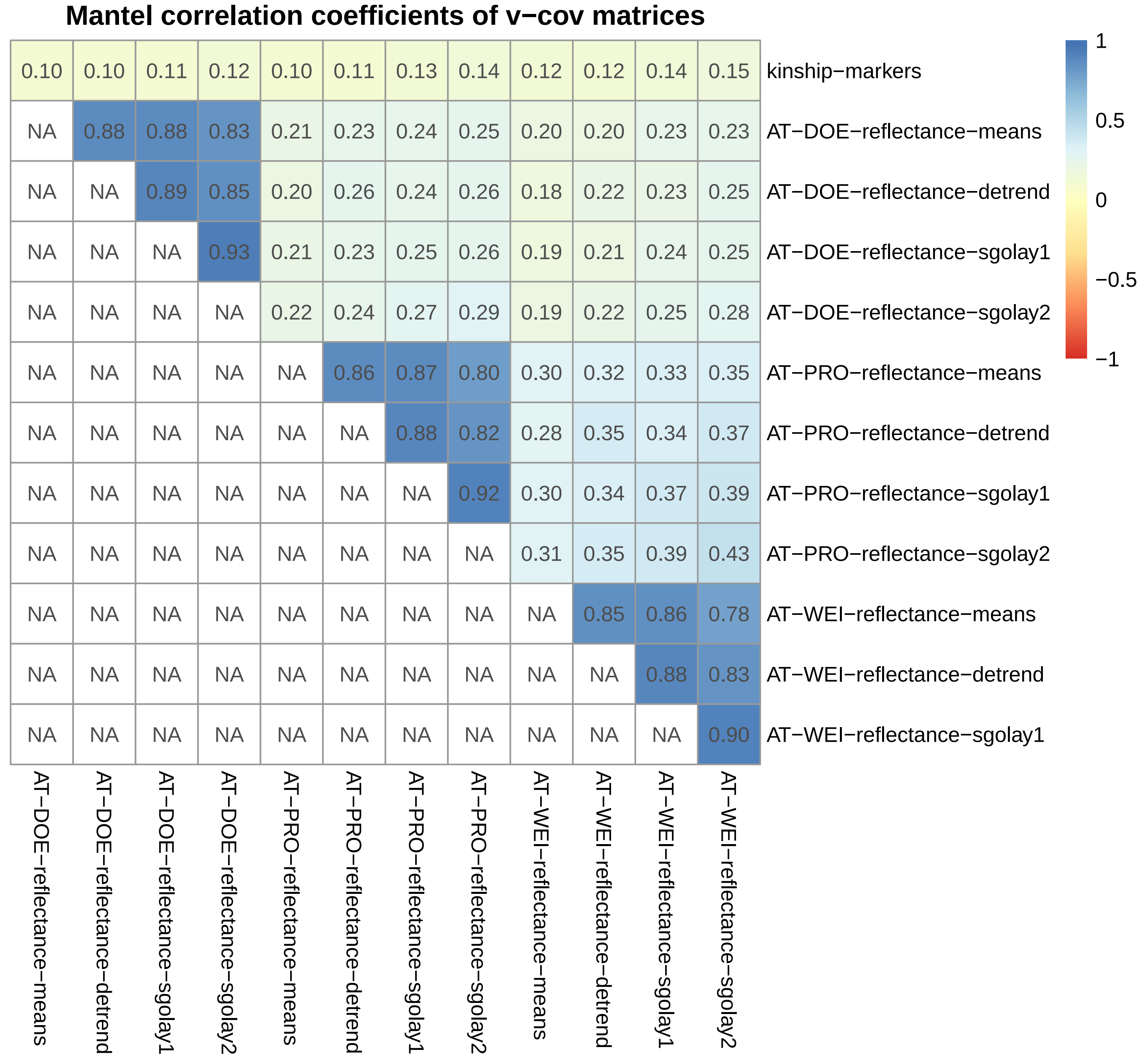
Mantel correlation coefficients of v−cov matrices
0.10 0.10 0.11 0.12 0.10 0.11 0.13 0.14 0.12 0.12 0.14 0.15 kinship−markers
NA 0.88 0.88 0.83 0.21 0.23 0.24 0.25 0.20 0.20 0.23 0.23 AT−DOE−reflectance−means
NA NA 0.89 0.85 0.20 0.26 0.24 0.26 0.18 0.22 0.23 0.25 AT−DOE−reflectance−detrend
NA NA NA 0.93 0.21 0.23 0.25 0.26 0.19 0.21 0.24 0.25 AT−DOE−reflectance−sgolay1
NA NA NA NA 0.22 0.24 0.27 0.29 0.19 0.22 0.25 0.28 AT−DOE−reflectance−sgolay2
NA NA NA NA NA 0.86 0.87 0.80 0.30 0.32 0.33 0.35 AT−PRO−reflectance−means
NA NA NA NA NA NA 0.88 0.82 0.28 0.35 0.34 0.37 AT−PRO−reflectance−detrend
NA NA NA NA NA NA NA 0.92 0.30 0.34 0.37 0.39 AT−PRO−reflectance−sgolay1
NA NA NA NA NA NA NA NA 0.31 0.35 0.39 0.43 AT−PRO−reflectance−sgolay2
NA NA NA NA NA NA NA NA NA 0.85 0.86 0.78 AT−WEI−reflectance−means
NA NA NA NA NA NA NA NA NA NA 0.88 0.83 AT−WEI−reflectance−detrend
NA NA NA NA NA NA NA NA NA NA NA 0.90 AT−WEI−reflectance−sgolay1
AT−DOE−reflectance−means
AT−DOE−reflectance−detrend
AT−DOE−reflectance−sgolay1
AT−DOE−reflectance−sgolay2
AT−PRO−reflectance−means
AT−PRO−reflectance−detrend
AT−PRO−reflectance−sgolay1
AT−PRO−reflectance−sgolay2
AT−WEI−reflectance−means
AT−WEI−reflectance−detrend
AT−WEI−reflectance−sgolay1
AT−WEI−reflectance−sgolay2
1
0.5
0
−0.5
−1

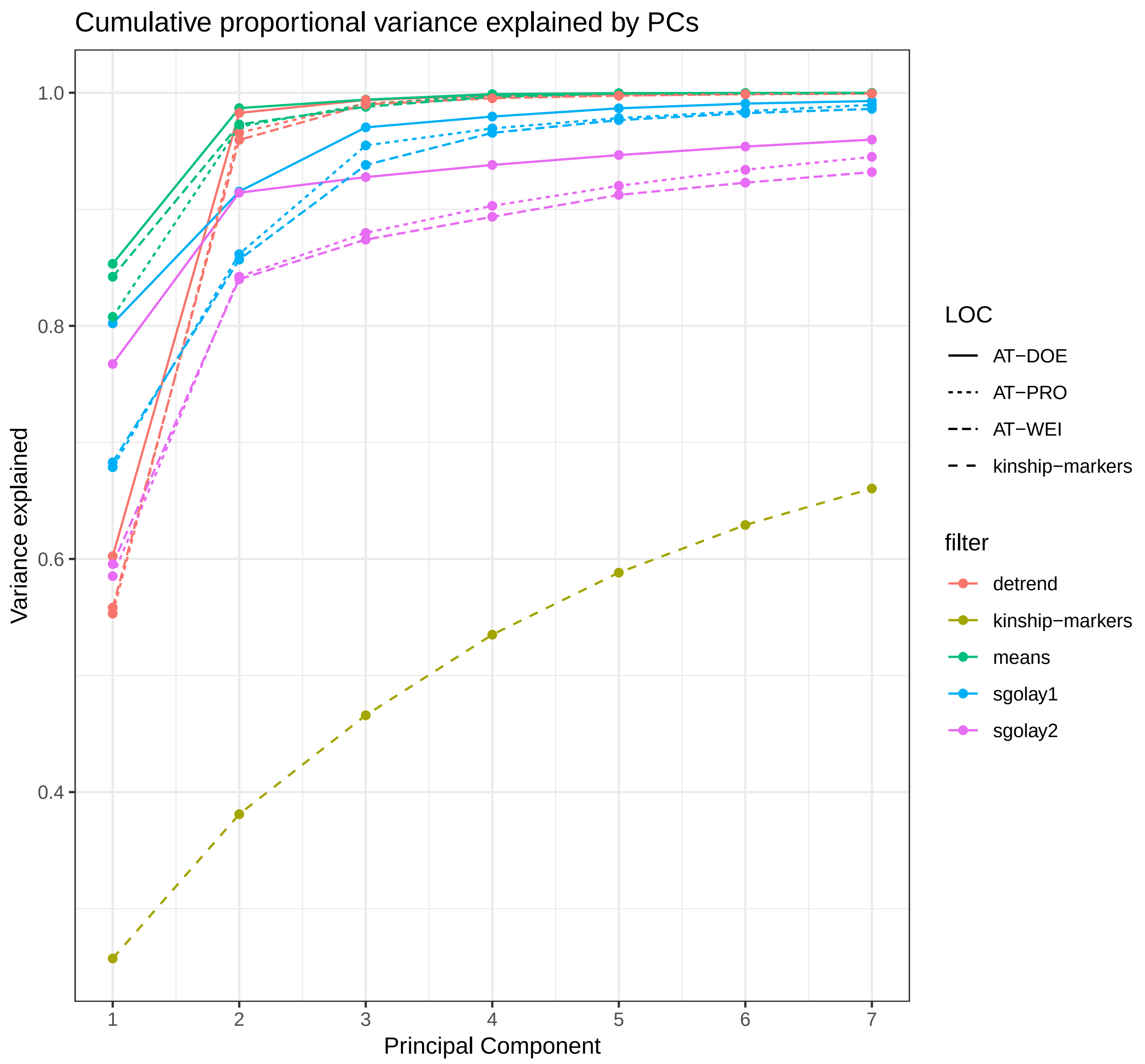
Cumulative proportional variance explained by PCs
Variance explained
1.0
0.8
0.6
0.4
Principal Component
1
2
3
4
5
6
7
LOC
AT−DOE
AT−PRO
AT−WEI
kinship−markers
filter
detrend
kinship−markers
means
sgolay1
sgolay2

Kinship estimated from markers

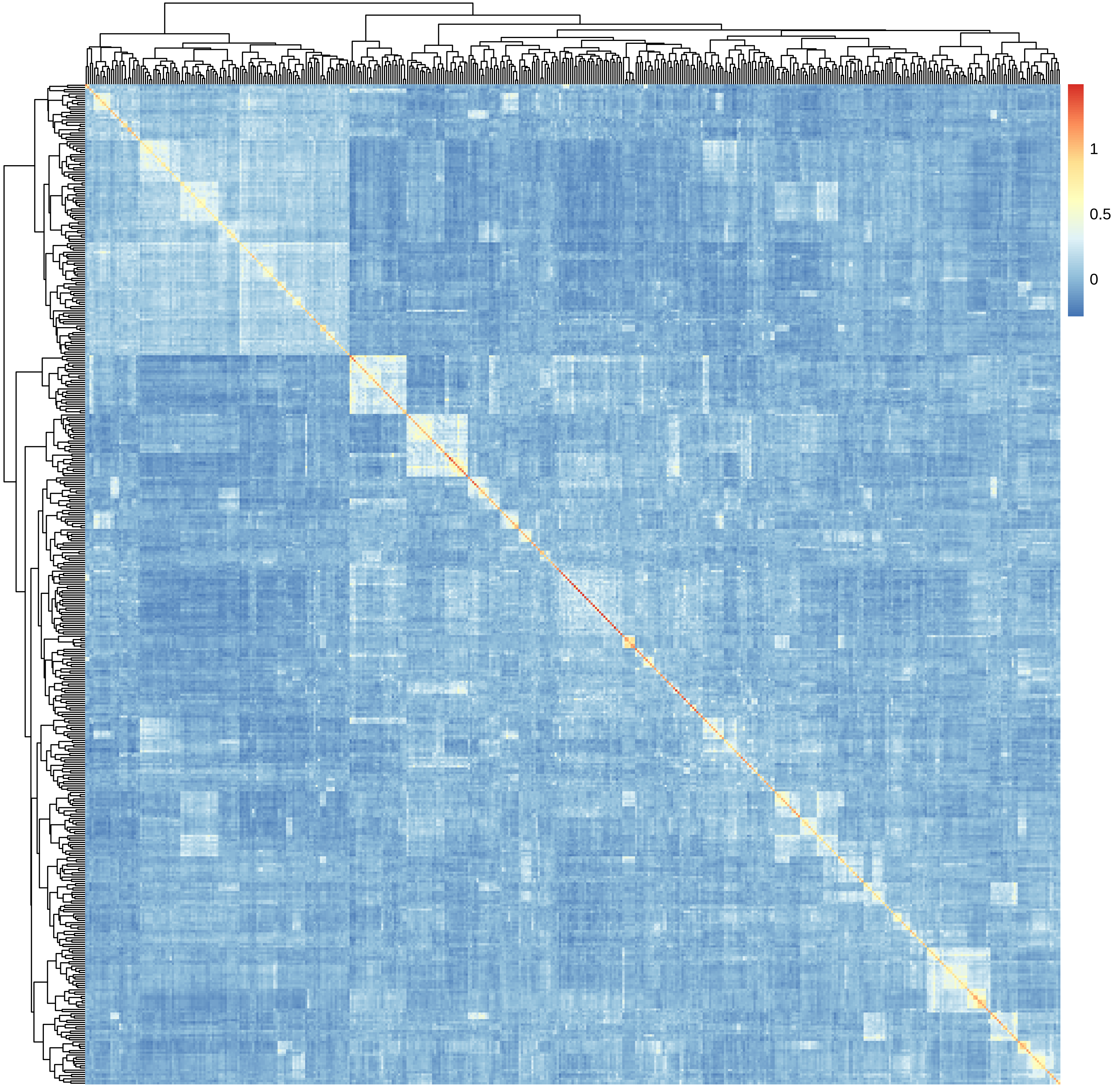

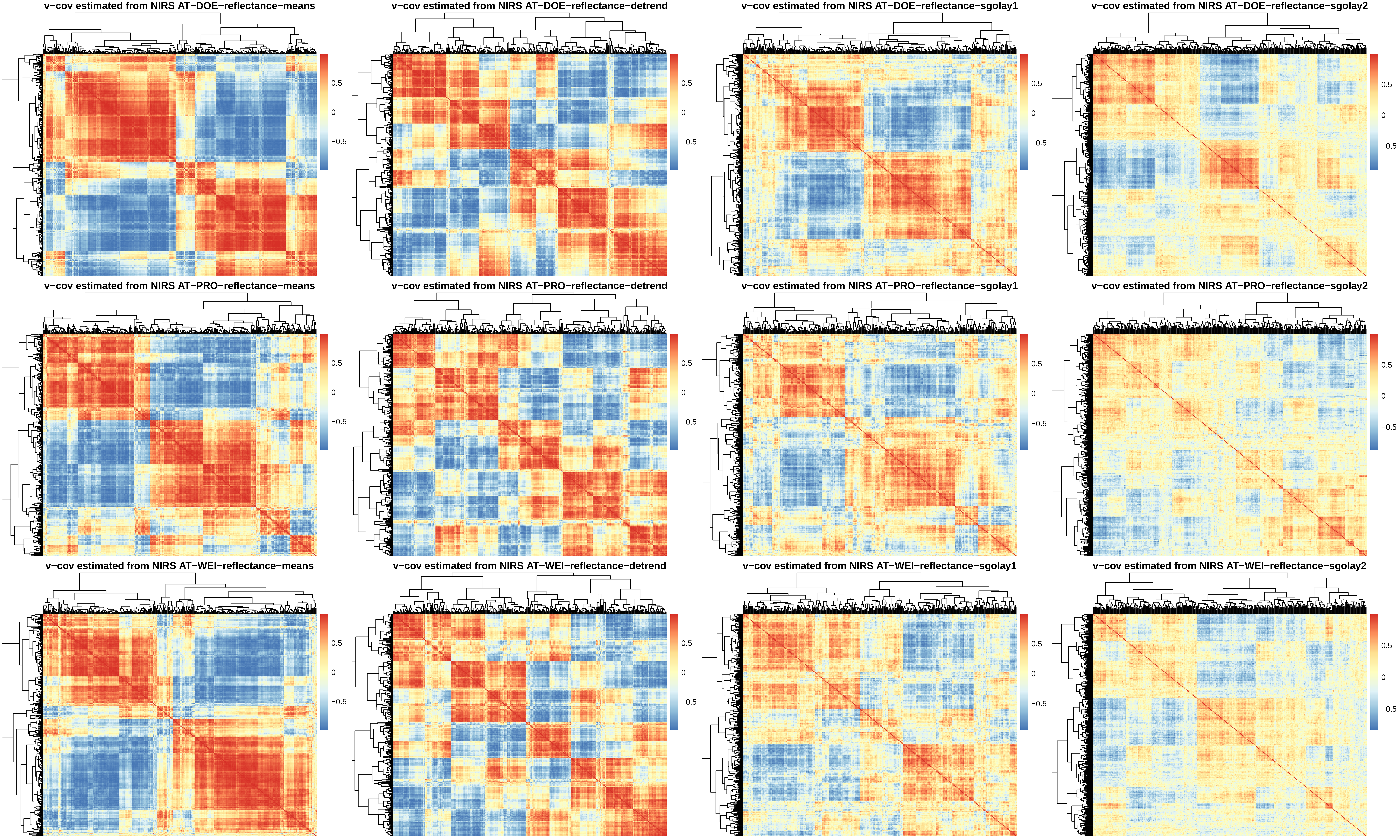
v−cov estimated from NIRS AT−DOE−reflectance−means
v−cov estimated from NIRS AT−DOE−reflectance−detrend
v−cov estimated from NIRS AT−DOE−reflectance−sgolay1
v−cov estimated from NIRS AT−DOE−reflectance−sgolay2
v−cov estimated from NIRS AT−PRO−reflectance−means
v−cov estimated from NIRS AT−PRO−reflectance−detrend
v−cov estimated from NIRS AT−PRO−reflectance−sgolay1
v−cov estimated from NIRS AT−PRO−reflectance−sgolay2
v−cov estimated from NIRS AT−WEI−reflectance−means
v−cov estimated from NIRS AT−WEI−reflectance−detrend
v−cov estimated from NIRS AT−WEI−reflectance−sgolay1
v−cov estimated from NIRS AT−WEI−reflectance−sgolay2
0.5
0
−0.5

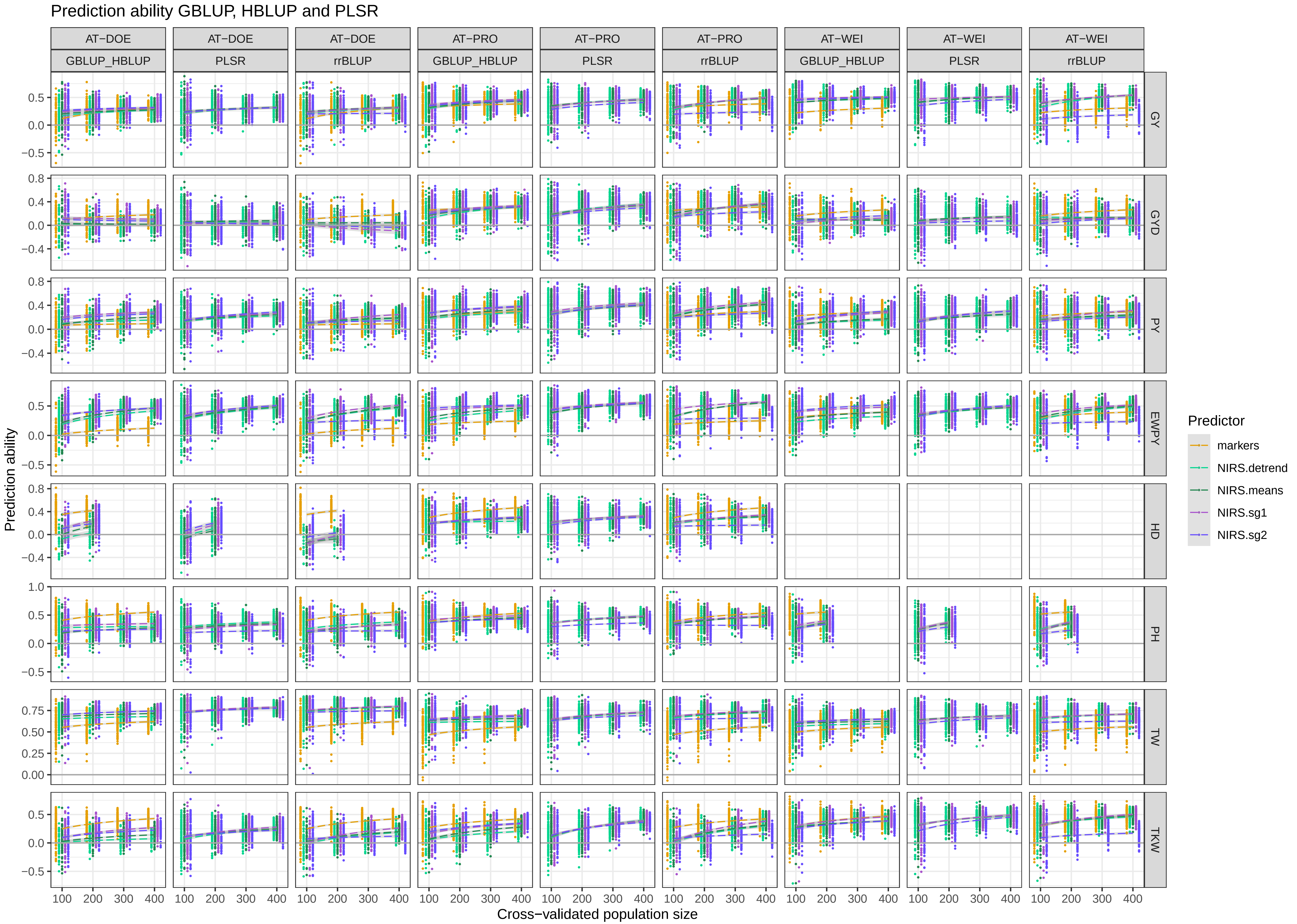
Prediction ability GBLUP, HBLUP and PLSR
AT−DOE
AT−PRO
AT−WEI
GBLUP_HBLUP
PLSR
rrBLUP
GY
GYD
PY
EWPY
HD
PH
TW
TKW
Prediction ability
Cross−validated population size
Predictor
markers
NIRS.detrend
NIRS.means
NIRS.sg1
NIRS.sg2

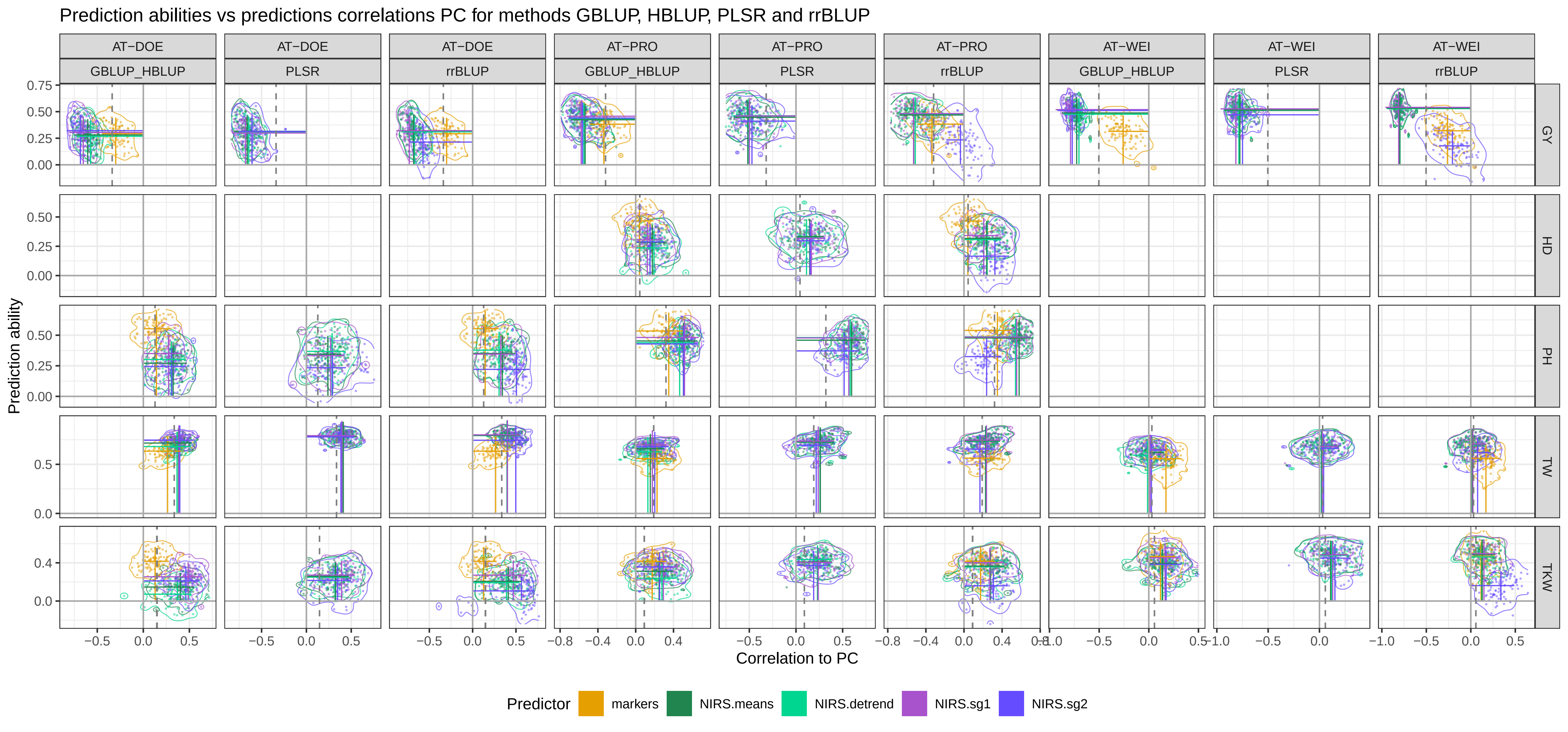

Prediction abilities vs predictions correlations PC for methods GBLUP, HBLUP, PLSR and rrBLUP
AT−DOE
AT−PRO
AT−WEI
GBLUP_HBLUP
PLSR
rrBLUP
GY
HD
PH
TW
TKW
Prediction ability
Correlation to PC
Predictor
markers
NIRS.means
NIRS.detrend
NIRS.sg1
NIRS.sg2

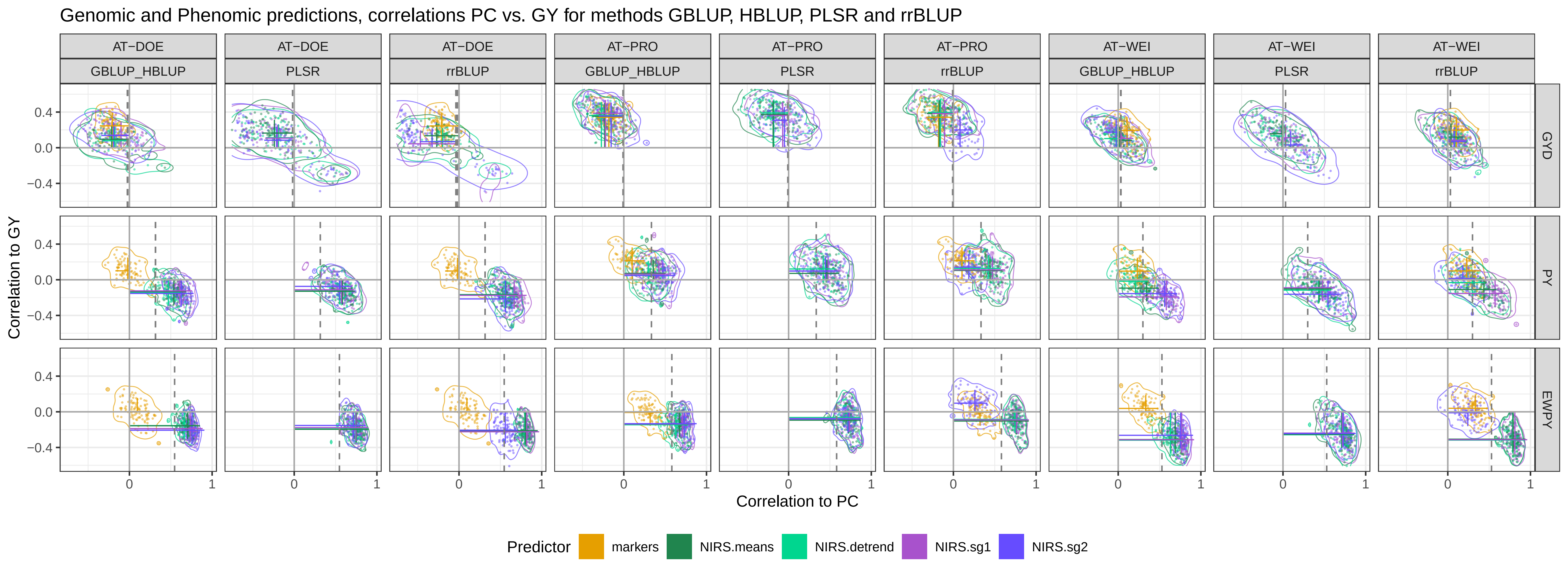

Genomic and Phenomic predictions, correlations PC vs. GY for methods GBLUP, HBLUP, PLSR and rrBLUP
AT−DOE
AT−PRO
AT−WEI
GBLUP_HBLUP
PLSR
rrBLUP
GYD
PY
EWPY
Correlation to GY
Correlation to PC
0.4
0.0
−0.4
0
1
Predictor
markers
NIRS.means
NIRS.detrend
NIRS.sg1
NIRS.sg2
